# Chloroplast genome sequence of the wild tetraploid potato relative *Solanum stoloniferum*

**DOI:** 10.1080/23802359.2018.1456983

**Published:** 2018-04-01

**Authors:** Tae-Ho Park

**Affiliations:** Department of Horticulture, Daegu University, Gyeongsan, South Korea

**Keywords:** Chloroplast, genome, genome sequence, *Solanum stoloniferum*

## Abstract

*Solanum stoloniferum* is a wild tuber-bearing species belonging to Solanaceae family. The complete chloroplast genome of *S. stoloniferum* was constituted by *de novo* assembly using a small amount of whole genome sequencing data. The chloroplast genome of *S. stoloniferum* was the circular DNA molecule with a length of 155,567 bp and consisted of 86,007 bp of large single copy, 18,374 bp of small single copy, and 25,593 bp of a pair of inverted repeat regions. A total of 158 genes were annotated including 105 protein-coding genes, 45 tRNA genes, and eight rRNA genes. Maximum likelihood phylogenetic analysis with 25 Solanaceae species revealed that *S. stoloniferum* is the most closely grouped with *S. tuberosum*.

*Solanum stoloniferum*, a wild tuber-bearing tetraploid species originating from Mexico is a relative to the cultivated potato, *S. tuberosum* (Hawkes 1990; Pendinen et al. 2008). It was identified to be a source of resistance to late blight and potato virus Y for potato breeding (Flis et al. 2005; Valkonen et al. 2008; Wang et al. 2008). However, the species is sexually incompatible with *S. tuberosum* due to different endosperm balance numbers (EBNs) with EBN values of 2 and 4 in *S. stoloniferum* and *S. tuberosum*, respectively, although both species are tetraploid (Brown 1988; Singsit and Hanneman 1991; Ortiz and Ehlenfeldt 1992; Cho et al. 1997). So, the wild species is difficult to be applied to potato breeding and somatic hybridization could be one of the solutions to overcome sexual barriers for interspecific gene transfer (Bidani et al. 2007; Nouri-Ellouz et al. 2016) and importance to identify chlorotype with information of chloroplast genome sequences in potato breeding program has increased (Chen et al. 2013, 2016; Cho and Park 2016; Cho et al. 2016; Molnár et al. 2017).

The *S. stoloniferum* (PI160224) was provided by Highland Agriculture Research Institute, South Korea. An Illumina paired-end (PE) genomic library was constructed with total genomic DNA according to the PE standard protocol (Illumina, San Diego, CA) and sequenced using an Illumina HiSeq2000 at Macrogen (http://www.macrogen.com/kor/). Low-quality bases with raw scores of 20 or less were removed and approximately 5.3 Gbp of high-quality of PE reads were assembled by a CLC genome assembler (CLC Inc, Arhus, Denmark) (Kim et al. 2015). The reference chloroplast genome sequence of *S. commersonii* (KM489054, Cho et al. 2016) was used to retrieve principal contigs representing the chloroplast genome from the total contigs using Nucmer (Kurtz et al. 2004). The representative chloroplast contigs were arranged in order based on BLASTZ analysis (Schwartz et al. 2003) with the reference sequence and connected to a single draft sequence by joining overlapping terminal sequences. DOGMA (Wyman et al. 2004) and BLAST searches were used to predict chloroplast genes.

The complete chloroplast genome of *S. stoloniferum* (GenBank accession no. MF471373) was 155,567 bp in length including 25,593 bp inverted repeats (IRa and IRb) regions separated by small single copy (SSC) region of 18,374 bp and large single copy (LSC) region of 86,007 bp with the typical quadripartite structure of most plastids, and the structure and gene features were typically identical to those of higher plants. A total of 158 genes with an average size of 584.4 bp were annotated including 105 protein-coding genes with an average size of 766.6 bp, 45 tRNA genes, and eight rRNA genes. An overall GC content was 37.87%.

Phylogenetic analysis was performed using chloroplast coding sequences of *S. stoloniferum* and 25 published species in Solanaceae family by a maximum likelihood method in MEGA 6.0 (Tamura et al. 2013). According to the phylogenetic tree, *S. stoloniferum* belonged to the same clade in *Solanum* species as expected and interestingly it was most closely grouped with *S. tuberosum* (Figure 1).

**Figure 1.**
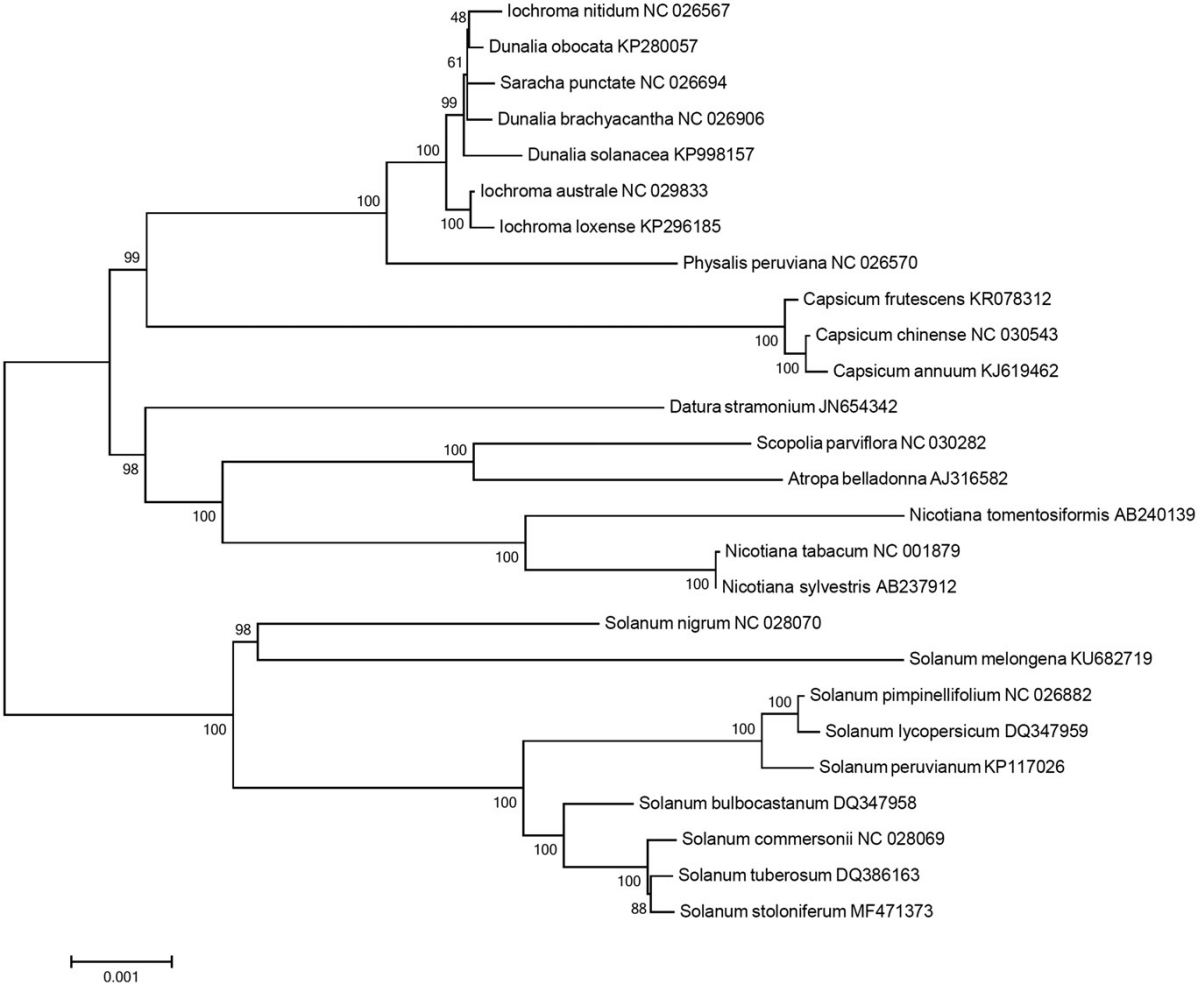
Maximum likelihood phylogenetic tree of *S. stoloniferum* with 25 species belonging to the Solanaceae based on chloroplast protein coding sequences. Numbers in the nodes are the bootstrap values from 1000 replicates.
